# Floral Color Properties of Serpentine Seep Assemblages Depend on Community Size and Species Richness

**DOI:** 10.3389/fpls.2020.602951

**Published:** 2021-01-08

**Authors:** Kathryn A. LeCroy, Gerardo Arceo-Gómez, Matthew H. Koski, Nathan I. Morehouse, Tia-Lynn Ashman

**Affiliations:** ^1^Department of Biological Sciences, University of Pittsburgh, Pittsburgh, PA, United States; ^2^Department of Environmental Sciences, University of Virginia, Charlottesville, VA, United States; ^3^Department of Biological Sciences, East Tennessee State University, Johnson City, TN, United States; ^4^Department of Biological Sciences, Clemson University, Clemson, SC, United States; ^5^Department of Biological Sciences, University of Cincinnati, Cincinnati, OH, United States

**Keywords:** community assembly, pollinator color vision, pollinator-mediated competition, floral color, cognitive pollination ecology

## Abstract

Functional traits, particularly those that impact fitness, can shape the ecological and evolutionary relationships among coexisting species of the same trophic level. Thus, examining these traits and properties of their distributions (underdispersion, overdispersion) within communities can provide insights into key ecological interactions (e.g., competition, facilitation) involved in community assembly. For instance, the distribution of floral colors in a community may reflect pollinator-mediated interactions between sympatric plant species, and the phylogenetic distribution of color can inform how evolutionary contingencies can continue to shape extant community assemblages. Additionally, the abundance and species richness of the local habitat may influence the type or strength of ecological interactions among co-occurring species. To evaluate the impact of community size and species richness on mechanisms shaping the distribution of ecologically relevant traits, we examined how floral color (defined by pollinator color vision models) is distributed within co-flowering assemblages. We modeled floral reflectance spectra of 55 co-flowering species using honeybee (*Apis mellifera*) and syrphid fly (*Eristalis tenax*) visual systems to assess the distributions of flower color across 14 serpentine seep communities in California. We found that phylogenetic relatedness had little impact on the observed color assemblages. However, smaller seep communities with lower species richness were more overdispersed for flower color than larger, more species-rich communities. Results support that competitive exclusion could be a dominant process shaping the species richness of flower color in smaller-sized communities with lower species richness, but this is less detectable or overwhelmed by other processes at larger, more speciose communities.

## Introduction

Competition for local resources like soil nitrogen and larger-scale factors such as climate have historically been documented as driving forces of plant community assembly (Webb, 2000; Fargione et al., 2003), but the persistence of a plant species in a community is contingent upon effective fertilization and seed production, which is mediated by animal pollinators for most angiosperms (Ollerton et al., 2011). Pollinators select flowers based on a variety of visual and olfactory cues and therefore have the potential to shape floral signal diversity in plant communities (Waser, 1986; Gumbert et al., 1999). Incorporating cognitive pollination ecology into plant community assembly studies is thus likely to prove fruitful for understanding the importance of plant–pollinator interactions and pollinator-mediated selection in flowering plant communities (Sargent and Ackerly, 2008; Schiestl and Johnson, 2013; Leonard and Masek, 2014; E-Vojtkó et al., 2020). In particular, there is a growing body of literature that has incorporated insights from pollinator vision to better understand the distribution of floral color in communities (de Jager et al., 2011; Dyer et al., 2012; Binkenstein et al., 2013; Shrestha et al., 2013; Burd et al., 2014; Muchhala et al., 2014; Makino and Yokoyama, 2015; Kemp et al., 2019; Shrestha et al., 2019).

Plant–pollinator interactions can shape the distribution of floral traits through their involvement in processes like competition or facilitation for visitation (Webb et al., 2002; Sargent and Ackerly, 2008). Such mechanisms may counteract or exacerbate abiotic processes such as habitat filtering (Ackerly, 2003) or stochastic processes such as neutral assembly (Hubbell, 2001). Ecological competition and facilitation are considered to operate at local spatial scales (Cavender-Bares et al., 2006). Examples of competitive exclusion shaping floral color assembly involve co-flowering plants competing for pollinators. In particular, selection may favor distinctiveness in floral coloration relative to other community members in a co-flowering assemblage, as this may aid in recognition by pollinators and support pollinator fidelity (Chittka, 1997; Gumbert et al., 1999; McEwen and Vamosi, 2010; Muchhala et al., 2014). This outcome would produce trait overdispersion of floral color (Sargent and Ackerly, 2008). Alternatively, facilitation may occur where one or more co-flowering species enhance another species’ reproductive success. A mechanism of facilitation involves one (or both) co-flowering species enhancing pollinator visitation to the other due to their high similarity in floral color, which can enhance perceived floral abundance or other attraction for pollinators more so than a single species could produce alone (Rathcke, 1983; Bruno et al., 2003; Moeller, 2004; Ghazoul, 2006). Habitat filtering or ecological facilitation may produce a pattern of trait underdispersion (clustering) at the local habitat scale, dependent upon phylogenetic constraint (Sargent and Ackerly, 2008). Alternatively, high similarity in floral color between species may be a product of Batesian mimicry, where an unrewarding co-flowering species offers sensory cues (e.g., floral color) highly similar to its co-flowering model, or Müllerian mimicry, where both flowering species offer rewards and collectively offer a greater advertising display of flower (Benitez-Vieyra et al., 2007). In addition, evaluating phylogenetic community structure is crucial for inferring ecological mechanisms producing trait community structure (Webb et al., 2002; Wolowski et al., 2017), as phylogenetic inertia can serve as a source of constraint on community assemblage (van der Niet and Johnson, 2012).

In studies to date, both overdispersion and underdispersion of floral color have been documented in flowering communities (e.g., overdispersion: Muchhala et al., 2014; Makino and Yokoyama, 2015; underdispersion: McEwen and Vamosi, 2010; Kemp et al., 2019). At local spatial scales where competition and facilitation are considered to occur, habitat availability (e.g., the amount of inhabitable area) and species richness of local habitat may influence the type and strength of ecological interactions observed in plant–pollinator community assemblage, but these factors have been understudied (Rathcke, 1983; E-Vojtkó et al., 2020). Understanding the impact that habitat availability and species richness has on pattern interpretation could provide better context for inference of these ecological mechanism(s) (Cavender-Bares et al., 2006; Kraft et al., 2007).

To investigate signatures of overdispersion or clustering in the distribution of flower color across communities with varying habitat availability and species richness, we studied the assemblages of co-flowering plant species in the serpentine seeps of northern California. Seeps are an excellent model for studying questions of community assembly due to their metacommunity structure and constricted window of flowering time (Harrison et al., 2000; Freestone and Harrison, 2006). With this metacommunity, we asked: (A) Are co-flowering assemblages more or less diverse in floral color as viewed by common flower visitors compared to random expectation? (B) Is there an effect of seep area, plant density, and plant species richness on the floral color trait dispersion as viewed by common flower visitors? (C) Is there phylogenetic structure in the co-flowering communities?

## Materials and Methods

### Study System

**FIGURE 1:**
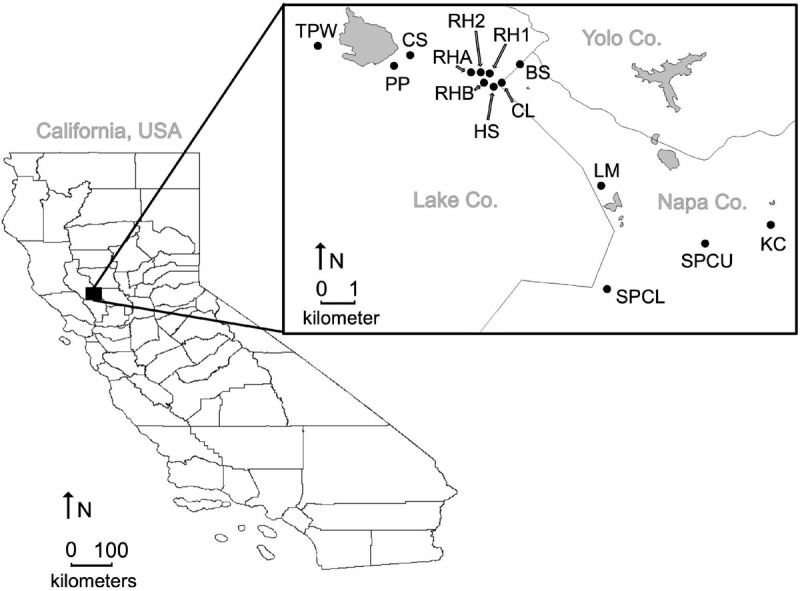
Spatial distribution of serpentine seep communities (black circles) at McLaughlin Natural Reserve in Lake and Napa Counties, California, United States. Gray polygons represent bodies of water.

We studied the floral assemblages of 14 serpentine seeps and the greater regional pool of co-flowering plant species at the McLaughlin Natural Reserve in northern California, United States (38°51′029.45″N, 122°24′033.49″W). In particular, seeps are tributaries of creeks and are characterized by the water that flows slightly below the ground surface, creating a wet soil environment for much of the dry season in northern California (Harrison et al., 2000). The unique plant communities of each seep occur within a matrix of chaparral and grassland, and seeps are composed of bare, rocky outcrops interspersed with more suitable microclimates for plant colonization and growth (Kruckeberg, 1984). Seeps in our study varied in area, from 0.04 to 0.55 km^2^, with an average area of 0.24 km^2^. Seeps were located at a minimum of 0.08 km and up to 13.57 km apart, with a mean distance of 4.24 km apart from one another (Figure 1). Surveys of co-flowering plant assemblages were conducted at each of the 14 seeps during June and July 2013, and these served as the “observed” local co-flowering species assemblages in our study. We also gathered floral spectra and insect visitation information about the broader “regional pool” of all co-flowering plant species across the entire serpentine seep metacommunity for use in constructing null models to compare with the observed communities (described in sections “Trait Community Structure Analyses” and “Phylogenetic Community Structure Analyses”). The inclusion of plant species in the broader regional pool was based on surveys performed in various seeps and immediately adjacent grassland in 2010, 2011, and 2013 (Alonso et al., 2013; Koski et al., 2015; Supplementary Table S1).

### Site Surveys

To observe local species assemblages to compare with the regional species pool, site surveys were performed once for each site in the peak seep flowering period spanning June and July of 2013. To estimate values of habitat availability, we estimated seep area as well as plant density in the seeps. Plant density at each site was evaluated to account for how much of the seep was habitable for plant colonization and growth compared to the amount of uninhabitable, rocky outcrops that also compose serpentine seeps (Kruckeberg, 1984; Wolf et al., 1999). To estimate seep area, seep length and average seep width were measured. A transect line was laid along the longest axis of each seep. The length of the seep was measured along this line, and the width of the seep was measured at five different points at roughly even increments along the length of the seep to find the average width of seep. Along the transect line of the seep length, a transect tape was placed in the center of the seep running parallel to the transect line. To estimate values of plant species richness and plant density, at every 0.25 m of the transect tape, we documented whether the tape was over bare ground (soil or rock) or in contact with a plant. If it was in contact with a plant, the plant was identified to species. Species richness was calculated as the number of different species occurring in the survey of each seep. Additionally, to survey plants growing at the edge of the seep that may prefer slightly different microhabitat than in the center of the seep outcrops, a transect line was run in parallel to the edge of the seep length either on the east or south side of the seep (depending on how the length of the seep was oriented in the field). The seep edge was surveyed in the same manner as the seep length transect line. Each site was surveyed in this manner for up to 100 m. If a seep was longer than 100 m, then 50 m of the survey was conducted at the ends of the longest axis of the seep (25 m at each end) and 50 m of the survey were conducted in the middle of the seep.

### Quantification of Seep Habitat: Area, Plant Density, and Species Richness

Seep area, plant density, and plant species richness within each seep were significantly and highly positively correlated (Supplementary Table S2), and we therefore consolidated these variables using principal component analysis (PCA). The first principal component, PC1, explained 75% of the variation in data. Negative loadings of PC1 indicated a small area, low plant density, and low species richness, whereas positive loadings of PC1 referred to larger, denser, and species-rich seep communities (Supplementary Table S3 and Supplementary Figure S1). We utilized PC1 as a summary that we define as “seep index.”

### Collection of Floral Spectra, Background Spectra, and Irradiance

Of 63 regional co-flowering species known to be present in seeps or directly adjacent to seeps, we collected reflectance spectra from 55 species (Supplementary Table S1 and Supplementary Figure S2). These reflectance spectra were collected during the 2012 and 2013 field seasons in the months of June and July. We measured floral reflectance spectra from five different individual plants for 45 species, four different individual plants for one species (*Plagiobothrys stipitatus*), three different individual plants for three species (*Acmispon parviflorus*, *Allium amplectans*, and *Antirrhinum cornutum)*, two measurements from different individual plants for two species (*Hesperolinon disjunctum* and *Mimulus layneae*), and one individual plant for four species (*Heterocodon rariflorum*, *Lagophylla minor*, *Sisyrinchium bellum*, and *Collinsia sparsiflora*). Of the 63 species in the regional pool, the following eight remaining species could not be measured for color: *Allium falcifolium*, *Calochortus luteus*, *Clarkia purpurea*, *Euphorbia* sp., *Lactuca saligna*, *Linanthus* sp., *Plantago erecta*, and *Triteleia laxa*. All eight species had been documented sporadically in seep communities and adjacent grassland in either 2010 or 2011 (unpublished data), and they were not recorded during seep surveys in 2013. We therefore exclude these eight species for community trait analysis and community phylogenetic analysis.

Across all reflectance spectra collected in 2012 and 2013, three spectrometers were utilized (USB2000+, USB4000, and Jaz, Ocean Optics, Dunedin, FL, United States; species-specific details in Supplementary Table S4). Spectra were collected using either an internal pulsed-xenon light source (Jaz, Ocean Optics, Dunedin, FL, United States) or a deuterium–halogen light source (DH-2000-BAL, Ocean Optics, Dunedin, FL, United States) with a Spectralon white standard (Labsphere, North Sutton, NH) and dark correction to measure percent reflectance from 300 to 700 nm, which is the general range of color perception by many flower-visiting insects, including bees and flies (Peitsch et al., 1992; Chittka, 1997). Floral tissue was illuminated with a collimated beam oriented normal to the floral surface, and spectra were collected by a probe positioned at a 45° azimuth, composed of a collimating lens and optical fiber (fiber diameter = 400 microns) connected to the spectrophotometer. We utilized SpectraSuite version 2.0.162 software for capturing spectral data (Ocean Optics, Dunedin, FL, United States). Spectra were collected with an integration time ranging from 50 to 250 ms and a boxcar smoothing width ranging from 3 to 25 nm, with a range of 10–30 average spectral scans (species-specific details of these parameters are included in Supplementary Table S4).

In collection of floral spectra, at least one single petal of the floral unit was measured for each flowering species, or in the instance that a single petal was too small to cover the entire sampling area, multiple petals were overlaid to provide enough surface for the spectrometer to collect a reflectance reading (McEwen and Vamosi, 2010). Within each floral unit, if there was a noticeable change in coloration in the human vision color spectrum or morphological component (e.g., petal vs. labellum), reflectance readings were obtained from various portions across the floral unit. We also searched for any change in ultraviolet reflectance range across the floral display area by viewing live spectrometer reflectance output while moving across the floral tissue surface. In total, 24 species of the 55 species were found to have variation in color within a given floral unit. Any noted differences within a floral unit were measured, and these details are included in Supplementary Information for each plant species (Supplementary Table S5).

To model floral visitor perception of floral colors under biologically relevant lighting conditions experienced during foraging, we measured solar irradiance at a single location at midday to represent study sites at midday (McLaughlin Natural Reserve Housing Site, Lower Lake, CA, United States: 38°52′23.82″N, 122°25′53.85″W) using a calibrated portable ultraviolet-visible (UV-vis) spectrophotometer (Ocean Optics JAZ, Ocean Optics, Dunedin, FL). Additionally, to represent a typical background against which floral colors were viewed by floral visitors, we measured the green foliage of five plant species occurring in the serpentine seep community (*H. rariflorum*, *Hoita macrostachya*, *Mimulus guttatus*, *Triteleia peduncularis*, and *Toxicoscordion venenosum*) using the same spectrometry techniques as applied to floral color measurement. We averaged these foliage spectra to produce a composite background reflectance spectrum.

### Processing of Spectral Data

Within each species, all floral reflectance spectra were averaged to produce representative floral reflectance spectra for each species. If spectral variation within the flowering unit was documented, these reflectances were weighted by the proportion of their representative measured area within a floral unit. This proportion was estimated by searching for distinct changes in UV spectral reflectance along the surface of the flower using the spectrometer (when considering UV internal contrast) or the percent area for each different color in human color perception was estimated by eye. The weighted spectral reflectances were then averaged together to create one reflectance reading for a given species. We chose to use this aggregate reflectance spectra because this represents the information available to floral visitors at typical foraging distances and is thus consistent with the information that might guide flower detection and visitation by insects (Lunau et al., 2006; however, see Garcia et al., 2018).

### Floral Visitor Vision Modeling

To identify the predominant floral visitors of the seep metacommunity and choose suitable insect vision models for modeling floral color, we evaluated data from a prior study documenting the insects visiting flowering species in seeps recorded in 2010 (Koski et al., 2015). From over 250 h of visitation observation in these seep communities, Koski et al. (2015) observed 15 functional groups of flower visitors to the seeps. Six of the 15 functional groups were of different groups of bees (large social bees, extra-large social bees, small solitary bees, medium solitary bees, large solitary bee species carrying pollen on legs, and large solitary bee species carrying pollen on body), and four functional groups were flies (Bombyliidae, large-size Syrphidae, small-size Syrphidae, and non-bombyliid/syrphid flies). The majority of flower visitation observed in the seeps were made by these 10 functional groups (Koski et al., 2015). From these findings, we then were able to identify the most common insect visitors, which were primarily bees (Hymenoptera), followed by flies (Diptera) (Supplementary Table S1).

To estimate the color appearance of flowers in our seep communities as viewed by their insect visitors, we used receptor noise-limited models of color vision for representative bee (*Apis mellifera*) and fly (*Eristalis tenax*) flower visitors (Vorobyev and Osorio, 1998). We utilized the European honeybee (*A. mellifera*) as our hymenopteran color vision model (Peitsch et al., 1992) because spectral sensitivity data are currently unavailable for the hymenopteran floral visitors endemic to these seep communities (Koski et al., 2015), and spectral sensitivities are largely conserved across Hymenoptera (Briscoe and Chittka, 2001). We also utilized a known syrphid fly color vision model, *E. tenax* (Horridge et al., 1975) because flies, including syrphid flies, were documented as the second-most common flower visitors to the serpentine seep plant community (Koski et al., 2015). Detailed equations of our visual system models are described in Supplementary Methods S1.

The European honeybee *A. mellifera* exhibits three color photoreceptor types: ultraviolet (UV), blue (B), and green (G) (Peitsch et al., 1992; Briscoe and Chittka, 2001). The photoreceptor types thought to be generally responsible for color perception in the syrphid fly *E. tenax* are ultraviolet (R7P), violet (R7Y), blue (R8P), and green (R8Y) *sensu* Ohashi et al. (2015). We used the known photoreceptor sensitivities of *A. mellifera* from Peitsch et al. (1992), and we used the known photoreceptor sensitivities of *E. tenax* provided by M. Shrestha and A. G. Dyer (personal communication, Shrestha et al., 2016). For both *A. mellifera* and *E. tenax* vision systems, we modeled the stimulation for all pairwise combinations of flowering species spectra in the regional pool against the collected green background foliage spectra under daylight illumination. This pairwise color disparity estimate between two floral spectra is termed Δ*S*^*t*^, measured in units of standard deviations of receptor noise between two color stimuli. With larger values of Δ*S*^*t*^, the two color stimuli are theorized to be more easily distinguishable by the viewer (as calculated using the equations provided in Supplementary Methods S1; Vorobyev and Osorio, 1998). The Δ*S*^*t*^ estimates for each pair of floral color spectra were computed with a script using NumPy 1.19.1 (Harris et al., 2020) in Python 3.8.5 (Van Rossum and Drake, 2009) following the methods outlined in Morehouse and Rutowski (2010).

To calculate Δ*S*^*t*^, photoreceptor noise and relative photoreceptor abundances for *A. mellifera* were incorporated into the model to estimate discriminability following Vorobyev and Osorio (1998). We chose to use a Weber fraction of 0.05 for the *A. mellifera* model, and the relative color photoreceptor abundances of *A. mellifera* were set as 2.125:1:9.375 for UV, B, and G photoreceptor types, respectively (Wakakuwa et al., 2005). Appropriate Weber fraction estimates and relative photoreceptor ratios are not known specifically for *E. tenax*. However, as with *A. mellifera*, we set out Weber fraction to 0.05 and used photoreceptor abundances known generally for flies as 1:2.33:1:2.33 for R7p, R7y, R8p, and R8y, respectively (Earl and Britt, 2006). We then mapped the relative stimulation outputs of each color photoreceptor type (for 55 flowering plant species) into a trichromatic color space for *A. mellifera* using the “ternaryplot” function in the vcd package version 1.4-8 (Meyer et al., 2020) and a tetrachromatic color space for *E. tenax* using the “colspace” function in the pavo package (Maia et al., 2019), both in R version 3.5.3 (R Core Team, 2019).

### Trait Community Structure Analyses

We compared the mean color disparity of each seep surveyed in 2013 (observed mean seep Δ*S*^*t*^) to the mean color disparity of 10,000 randomly assembled communities per observed seep, termed null mean seep Δ*S*^*t*^. For each of the 14 surveyed (observed) seeps, we generated 10,000 randomly assembled communities of species, with the species richness of the assembled communities limited to the species richness of the observed seeps. For example, if an observed seep in 2013 was found to have six co-flowering species, then it would be compared to 10,000 randomly assembled communities, each composed of six species. Within each of the 10,000 iterations of random community generation, species were drawn from the regional pool of the 55 co-flowering species without replacement. The mean Δ*S*^*t*^ for each randomly assembled community was estimated, and then a grand mean Δ*S*^*t*^ was calculated from all 10,000 communities, thereby creating the null mean seep Δ*S*^*t*^. This null mean seep Δ*S*^*t*^ was then used for comparison to the observed mean seep Δ*S*^*t*^ for each of the 14 observed seep communities. These randomly assembled communities and estimation of null mean seep Δ*S*^*t*^ values were computed for each pollinator vision system with a script using NumPy 1.19.1 (Harris et al., 2020) in Python 3.8.5 (Van Rossum and Drake, 2009) following the methods outlined in Morehouse and Rutowski (2010).

Analyses of species assemblages restricted to those known to be visited by specifically bees or specifically flies may offer a more functionally relevant and conservative approach for understanding what each flower visitor might experience when foraging in a given seep community. Therefore, we also performed calculations of observed mean Δ*S*^*t*^ and null mean Δ*S*^*t*^ values that were restricted to (a) only the plant species recorded as visited by bees or (b) only the plant species recorded as visited by flies (Supplementary Table S1).

To test if (a) the observed mean seep Δ*S*^*t*^ values significantly differed from random expectations (null mean Δ*S*^*t*^ values) and if (b) observed mean seep Δ*S*^*t*^ depended upon seep index, we used analysis of covariance (ANCOVA) (SAS 9.4, PROC GLM). We modeled mean Δ*S*^*t*^ in a community as a function of community type (observed mean seep or null mean seep from 10,000 randomly generated communities) and seep index (the PC1 of observed seep area, plant density, and species richness). These analyses were performed in SAS 9.4 (SAS, 2014). A significant effect of community type suggests that flower color is either overdispersed or underdispersed. A relationship between seep index and trait distribution (mean Δ*S*^*t*^) is determined by the significance of the interaction term in the model. We ran these analyses for all four scenarios: bee vision system with all plant species, bee vision system with only bee-visited species, fly vision system with all plant species, and fly vision system with only fly-visited species. We also calculated z-scores for each observed community mean Δ*S*^*t*^ in comparison with its null model community mean Δ*S*^*t*^ for all four scenarios. For each ANCOVA, we inspected the normalized residuals of each model for any spatial autocorrelation using bubble plots, correlograms, variograms, and calculation of Moran’s *I*.

### Phylogenetic Community Structure Analyses

To evaluate phylogenetic community structure, an ultrametric phylogenetic tree of the regional species pool was constructed including all 55 species for which color was collected (Supplementary Figure S2). This was done by using Phylomatic 3.0 and Phylocom 4.2, which incorporated known branch lengths from Wikstrom et al. (2001) using the BLADJ function in Phylocom (2001).

To investigate any patterns of phylogenetic community structure within seep communities that would contextualize evidence for ecological mechanism, we calculated the observed mean phylogenetic distances (MPDs) for each of the seeps surveyed. This observed seep-specific metric was compared against the mean MPD measured from 10,000 random null communities generated for each seep, holding species richness constant but generating communities with random species from the regional (metacommunity) species pool. In effect, this function generates 10,000 random communities and compares the mean pairwise phylogenetic distances for each random community to the observed community.

To test whether (a) observed phylogenetic structure significantly differed from random expectations and whether (b) phylogenetic community structure is related to seep index, we used ANCOVA (SAS, PROC GLM). We modeled MPD as a function of community type (observed vs. null), seep index, and the interaction between community type and seep index in SAS 9.4 (SAS, 2014). A significant difference between observed mean MPD and null community mean MPD values supports overdispersion or underdispersion is present in phylogenetic structure. A significant interaction term in the model will test for significance of any relationship between seep index and phylogenetic overdispersion or underdispersion. For this ANCOVA, we inspected the normalized residuals for any spatial autocorrelation using bubble plots, correlograms, variograms, and calculation of Moran’s *I*.

## Results

### Trait Community Structure

The mean number of plant species recorded per seep was 14 species per seep, with a range of six to 20 species. Of these observed seep communities, an average of approximately 12 plant species per seep was known to be visited by bees (ranging from 5 to 17 species), and an average of approximately seven plant species per seep was known to be visited by flies (with a range from 2 to 13 plant species). For the fly-specific visitor community structure analysis, we chose to exclude the two seep communities that each only had two plant species known to be visited by flies, which were seep SPCL and seep SPCU.

**FIGURE 2:**
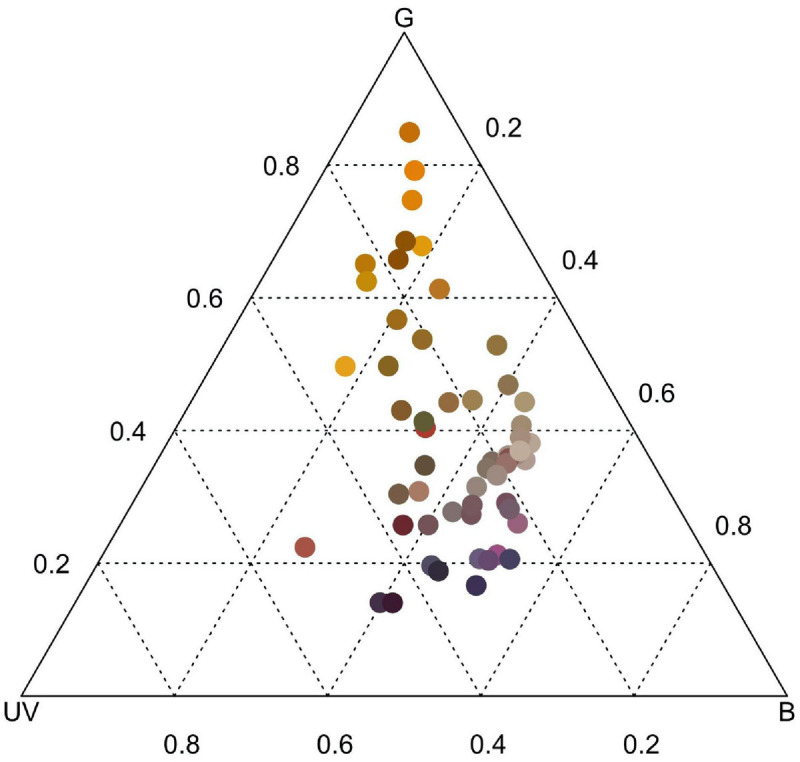
Bee color space depicting the differential stimulation of color photoreceptor types of *Apis mellifera* with all 55 co-flowering species from serpentine seep community. Color of circles represents human-perceived color of flowering structure for each species.

**FIGURE 3:**
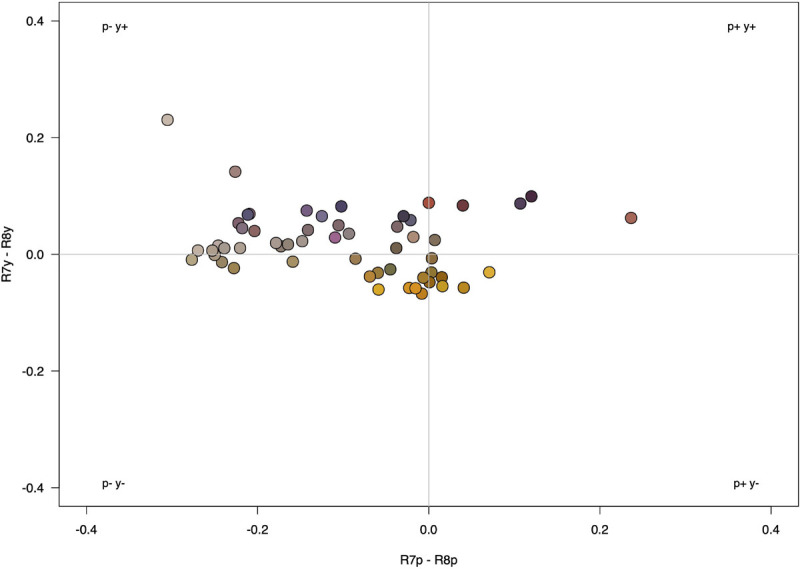
Fly color space depicting differential stimulation of color photoreceptor types of *Eristalis tenax*. Color of circles represents human-perceived color of flowering structure for each species.

**FIGURE 4:**
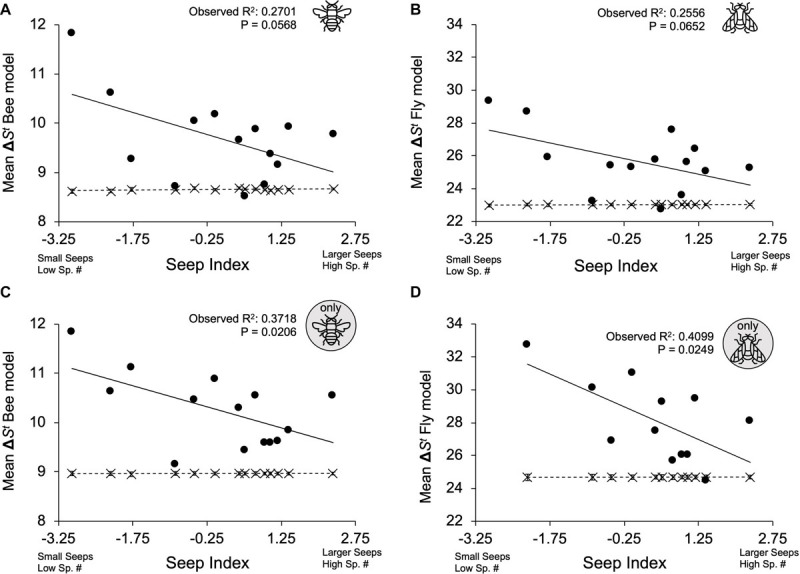
Comparison of observed and null mean Δ*S*^*t*^ values across seep index. Observed seep values are represented by black circles (⚫) and solid lines; randomly generated null communities are represented by “X” markers and dashed lines. Low seep index values refer to small seeps with low species richness, high seep index values correspond to large seeps with high species richness. Error bars present on null mean Δ*S*^*t*^ values represent 95% confidence intervals. **(A)** All co-flowering species modeled through bee vision system. **(B)** All co-flowering species modeled through fly vision system. **(C)** Model restricted to bee-visited plant species. **(D)** Model restricted to fly-visited plant species.

When floral color disparity (mean Δ*S*^*t*^) among all co-flowering species was modeled using the selected bee color vision model (Figure 2) and fly color vision model (Figure 3), on average, observed mean seep Δ*S*^*t*^ was overdispersed in observed communities, exhibiting significantly higher color differences compared to null model predictions [bee model, Figure 4A: *F*_(__3,__24__)_ = 25.53, *P* < 0.0001; observed mean Δ*S*^*t*^: 9.698, 95% CI (9.398, 9.998); null mean Δ*S*^*t*^: 8.660, 95% CI (8.360, 8.960); fly model, Figure 4B: *F*_(__3,__24__)_ = 34.18, *P* < 0.0001; observed mean Δ*S*^*t*^: 25.677, 95% CI (25.019, 26.339); null mean Δ*S*^*t*^: 23.028, 95% CI (22.366, 23.689)].

The bee model analysis with all plant species revealed a significant interaction between community type (observed vs. null) and seep index [*F*_(__3,__24__)_ = 4.35, *P* = 0.0477], with the observed mean Δ*S*^*t*^ declining with seep index (*t* = −2.09, *P* = 0.0477) but no relationship between the null mean Δ*S*^*t*^ and seep index (*t* = −0.03, *P* = 0.9769) (Figure 4A). The fly model with all plant species revealed a marginally significant interaction between community type (observed vs. null) and seep index [*F*_(__3,__24__)_ = 4.21, *P* = 0.0511], with the observed mean Δ*S*^*t*^ exhibiting a marginally significant decline with seep index (*t* = −2.05, *P* = 0.0511) with no relationship between the null mean Δ*S*^*t*^ and seep index (*t* = 0.03, *P* = 0.9740) (Figure 4B). No spatial structure was found in the model residuals for bee nor fly models with all plant species (bee model: Moran’s *I* = −0.1075, *P* = 0.7485; fly model: Moran’s *I* = 0.0482, *P* = 0.1869).

For the model that restricted the plant community to plant species known to be visited by bees, observed mean Δ*S*^*t*^ values calculated with the bee color vision model were significantly overdispersed compared to null model predictions [Figure 4C; *F*_(__3,__24__)_ = 48.50, *P* < 0.0001; observed mean Δ*S*^*t*^: 10.180, 95% CI (9.926, 10.434); null mean Δ*S*^*t*^: 8.968, 95% CI (8.714, 9.222)]. Additionally, there was a significant interaction between mean community type (observed vs. null) and seep index for the bee-visited community [*F*_(__3,__24__)_ = 7.14, *P* = 0.0133], and this interaction was driven by a decline in the observed mean Δ*S*^*t*^ with seep index (*t* = −2.67, *P* = 0.0133) but not by the null mean Δ*S*^*t*^ (*t* = 0.01, *P* = 0.9917) (Figure 4C).

For the model that restricted the plant community to plant species known to be visited by flies, mean Δ*S*^*t*^ values calculated with the fly color vision model were significantly overdispersed compared to null model predictions [Figure 4D; *F*_(__3,__2__0__)_ = 42.36, *P* < 0.0001; observed mean Δ*S*^*t*^: 28.105, 95% CI (27.260, 28.950); null mean Δ*S*^*t*^: 24.685, 95% CI (23.840, 25.530)]. There was also a significant interaction between community type (observed vs. null) and seep index for the fly-visited plant community (observed vs. random *F*_(__3,__2__0__)_ = 6.99, *P* = 0.0156), and this interaction was again driven by a decline in the observed mean Δ*S*^*t*^ with seep index (*t* = −2.64, *P* = 0.0156) but not the null mean Δ*S*^*t*^ (*t* = 0.01, *P* = 0.9918) (Figure 4D). No spatial structure was found in the model residuals for bee nor fly models restricted specifically by known visitation (bee-specific model: Moran’s *I* = 0.0108, *P* = 0.3431; fly-specific model: Moran’s *I* = −0.1290, *P* = 0.7056).

All z-scores for each observed mean seep Δ*S*^*t*^ compared to null mean seep Δ*S*^*t*^ are found in Supplementary Table S6 (bee model with all plant species), S7 (fly model with all plant species), S8 (bee model with bee-visited plant species), and S9 (fly model with fly-visited plant species).

### Phylogenetic Community Structure

**FIGURE 5:**
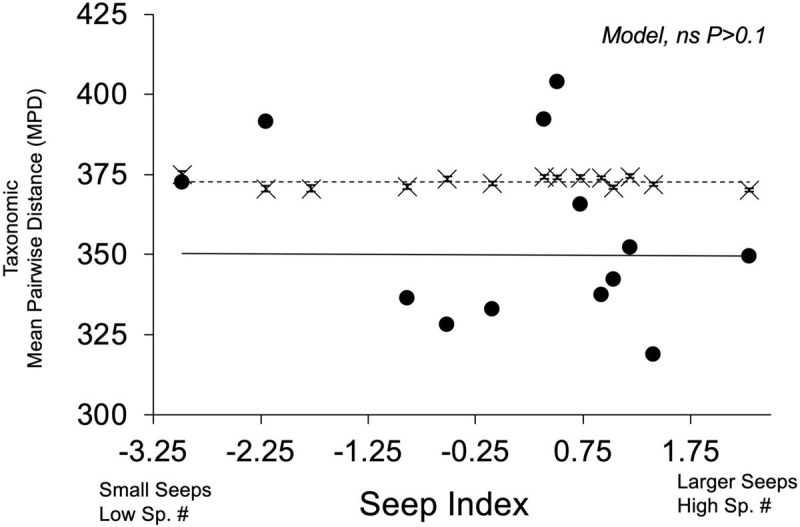
Comparison of observed and null mean phylogenetic distances (MPDs) across seep index. Observed seep values are represented by black circles (⚫) and solid line; randomly generated null communities are represented by “X” markers and dashed line. Error bars present on null MPD community values represent 95% confidence intervals.

There was no phylogenetic community structure detected in the observed seep communities, and there was no relationship between seep index and phylogenetic relatedness [*F*_(__3,__24__)_ = 1.91, *P* = 0.1542] (Figure 5). Seep-specific z-scores are found in Supplementary Table S10. No spatial structure was found in the model residuals (Moran’s *I* = −0.0781, *P* = 0.9898).

## Discussion

Observed assemblages of flower color in serpentine seep communities are overdispersed compared to random assemblages when viewing floral colors through models of both bee vision and fly vision. Flower color overdispersion was particularly strong when evaluating the two subsets of plants documented to be visited by bees and flies. Distinctiveness in floral color perception of a given species relative to other co-flowering community members could aid in recognition by pollinators, increase pollinator visitation rates, and support pollinator fidelity (McEwen and Vamosi, 2010; Muchhala et al., 2014). In particular, in the absence of phylogenetic structure, the overdispersion of a functional trait may indicate pollinator-mediated competition (Sargent and Ackerly, 2008). Our study did not evaluate species-specific pollination efficiency by floral visitors but instead considered floral appearance to presumed pollinators. If our assumption of visitors as pollinators holds, then our data support that the observed trait overdispersion is likely the result of ecological mechanisms rather than phylogenetic sorting because we failed to find a nonrandom phylogenetic community structure (Figure 5). However, our study did not evaluate instances of character displacement, which could also drive floral trait divergence by natural selection (Sargent and Ackerly, 2008).

In addition to competition for pollinator visits, another mechanism that could produce such a pattern of competitive exclusion and maintain overdispersion in visitor-perceived color is the avoidance of interspecific pollen transfer. Interspecific pollen transfer has been found to negatively impact plant reproductive success, including seed production, when pollinators are shared among plant species (Feinsinger et al., 1988; Morales and Traveset, 2008; Ashman and Arceo-Gómez, 2013), with interspecific pollen transfer involving both the loss of conspecific pollen transfer and the deposition of heterospecific pollen (Wilcock and Neiland, 2002; Mitchell et al., 2009). If community membership is structured to maximize pollinator-perceived color disparity, then plant coexistence and persistence can occur with limited negative impacts due to high pollinator fidelity and pollinator recognition (Chittka, 1997; McEwen and Vamosi, 2010). However, for any species immigrating into a community for which it overlaps with already established species in trait space, asymmetrical competition might occur through heterospecific pollen deposition and reduced conspecific pollen transfer (Runquist and Stanton, 2013). However, these predictions should be further experimentally evaluated in a community context, as interactive effects are known to mediate the magnitude of detrimental effects of heterospecific pollen transfer and deposition (Arceo-Gómez and Ashman, 2011; Arceo-Gómez et al., 2019).

Overdispersion of visitor-perceived color disparity was greater at smaller seeps with fewer species (Figures 4A–D). Competition may be more intense in small communities with fewer species because of reduced overall visitation by pollinators (Sargent and Ackerly, 2008). When shared pollinators do visit, there would be the risk of heterospecific pollen transfer for more similarly perceived co-flowering species as described previously. This overdispersion of color disparity decreased with greater seep habitat area and species richness (Figures 4A–D), which could suggest that competitive exclusion is a dominant ecological mechanism structuring flower color disparity in smaller, less species-rich seep communities, but this signature of competition may be either less detectable or not occurring in larger habitats. The lack of trait overdispersion in larger communities could be caused by other ecological mechanisms (such as facilitation or habitat filtering) drowning out competitive signals in seeps with a larger habitat area and greater species richness. In particular, this trend toward reduced overdispersion may be due to differences in the shifting dominance from biotic to abiotic mechanisms with increasing species richness and habitat area, regardless of phylogenetic community structure (Arista et al., 2013). Alternatively, lower seep mean Δ*S*^*t*^ could be the result of increased occupancy in color vision trait space exhibited in larger seeps with greater species richness; with larger community assemblages, the more trait values must fit within the same confines of trait space, resulting in higher packing density of color traits. However, further work in a field setting should be done to measure reproductive success of different co-flowering species with experimental manipulations of floral visitor exclusion, such as bees only, flies only, or both visitors in pollination assays.

Other studies have also detected overdispersion of floral color in co-flowering communities using metrics of insect color vision (Muchhala et al., 2014; Makino and Yokoyama, 2015), and our findings are consistent with previous studies finding evidence for competitive exclusion in pollinator color vision space regardless of phylogenetic community structure (Muchhala et al., 2014). Yet other studies have found that floral color is clustered when using pollinator vision models (Kemp et al., 2019) when using other colorimetric analyses independent of pollinator perception (McEwen and Vamosi, 2010). Further, Shrestha et al. (2019) found color assemblages that did not significantly differ from random expectation. Some studies have found evidence for ecological mechanisms at the level of plant clade (Muchhala et al., 2014; Briscoe Runquist et al., 2016) and plant family (Kemp et al., 2019). By studying 14 co-flowering assemblages with varying family associations, habitat availability, and species richness, our findings demonstrate that the detection of patterns such as trait overdispersion may depend on community size and species richness, and therefore, inference of mechanism may vary at these local scales.

Our study is limited by its focus on only one sensory modality involved in insect flower visitor behavior, namely, vision. Consideration of the role of other important sensory elements (e.g., olfactory cues, flower display size, floral reward composition) would allow a more complete understanding of how a community collectively attracts floral visitors and how the resulting interactions shape species presence and persistence in these seep habitats (Primante and Dötterl, 2010; Leonard et al., 2011; Junker et al., 2013; Wei et al., 2020). In addition, behavioral validation of our visual system modeling would help to critically test the role of pollinator–plant interactions in community assemblage implicated in our work. However, due to the relatively high mean Δ*S*^*t*^ community values in observed seeps for both bee and fly models, we predict that behavioral observations would confirm that these estimates of Δ*S*^*t*^ reflect accurate behavioral discrimination among co-flowering species (Fleishman et al., 2016; but see Garcia et al., 2018).

Other factors not considered in this study include an assessment of co-flowering species densities; within co-flowering communities, conspecific and heterospecific plant densities impact their individual reproductive success (Benadi and Pauw, 2018). We were also unable to document the floral color of eight plant species known to occur in this serpentine seep community, in part, because they were infrequently observed, and they were not observed at all in 2013 site surveys. Their rarity may impact the discriminability of the community that we have not been able to measure. We predict that their densities are low in seep areas where they do occur, but how density-dependent plant–pollinator interactions are mediated with changing habitat size and community species richness is an active area of theoretical and empirical study (Mesgaran et al., 2017; Benadi and Pauw, 2018; Bergamo et al., 2020).

In a rapidly changing world threatened with major losses of biodiversity and ecosystem function (Mori et al., 2013), ecologists must seek to elucidate the mechanisms that generate and sustain variation in observed patterns of co-occurring species or trait assemblages (Diamond, 1975). We have shown the evidence of small-scale community structuring in color discriminability in replicated communities, and we have provided evidence that these observed plant communities could be responding to flower visitors as selective forces in community assembly.

## Data Availability Statement

The datasets presented in this study can be found in the Supplementary Material, with the exception of spectral color data, which can be accessed in an online repository at this link: https://doi.org/10.5061/dryad.v41ns1rtq.

## Author Contributions

T-LA, GA-G, and KL conceived the study. NM trained KL on color vision modeling and interpretation of model results. GA-G, MK, and KL acquired floral specimens for spectral collection. KL conducted field surveys in 2013, led the writing of the manuscript, statistical analyses, and interpretation of community assembly results. All authors contributed critically to the drafts and gave final approval for publication.

## Conflict of Interest

The authors declare that the research was conducted in the absence of any commercial or financial relationships that could be construed as a potential conflict of interest.

## Supplementary Material

Supplementary Figure 1 Results of principal component analysis. **(A)** Proportion of variation explained by each Principal Component axis, with dashed line representing cumulative proportion, solid line representing proportion. **(B)** Eigenvalues of Principal Component Axes.

Supplementary Figure 2 Phylogenetic tree of 55 plant species used in study. Circles are colored to represent human vision interpretation of flower color. Branch length reference unit of 10 represents 10 Mya.

Supplementary Table 1 List of 55 plant species used in study, along with community membership and flower visitor information. Seep sites are ordered by increasing species richness. An X in the “F” Visit column denotes that a focal plant species is visited by fly species, an X in the “B” Visit column denotes that a focal plant species is visited by bee species.

Supplementary Table 2 Correlation panel for species richness, seep area, and plant density.

Supplementary Table 3 principal component loadings for species richness, seep area, and plant density.

Supplementary Table 4 Details of spectra collection parameters for each plant species.

Supplementary Table 5 Descriptions of noted color differences within the flowering unit of each plant species in the study.

Supplementary Table 6 Estimated z-scores of each observed seep mean Δ*S*^*t*^ compared to null community mean Δ*S*^*t*^ for bee vision model using all plant species regardless of known insect visitation.

Supplementary Table 7 Estimated z-scores of each observed seep mean Δ*S*^*t*^ compared to null community mean Δ*S*^*t*^ for fly vision model using all plant species regardless of known insect visitation.

Supplementary Table 8 Estimated z-scores of each observed seep mean Δ*S*^*t*^ compared to null community mean Δ*S*^*t*^ for bee vision model only using plant species known to be visited by bees.

Supplementary Table 9 Estimated z-scores of each observed seep mean Δ*S*^*t*^ compared to null community mean Δ*S*^*t*^ for fly vision model only using plant species known to be visited by flies.

Supplementary Table 10 Estimated z-scores of each observed seep taxonomic Mean Pairwise Distance (MPD) compared to null community taxonomic MPD.

Supplementary Methods 1 Detail of color vision modeling equations used in methods.

## References

[B1] Ackerly D. D. (2003). Community assembly, niche conservatism, and adaptive evolution in changing environments. *Int. J. Plant Sci.* 164 S165–S184. 10.1086/368401

[B2] Alonso C. Navarro-Fernández C. M. Arceo-Gómez G. Meindl G. A. Parra-Tabla V. Ashman T.-L. (2013). Among-species differences in pollen quality and quantity limitation: implications for endemics in biodiverse hotspots. *Ann. Bot.* 112 1461–1469. 10.1093/aob/mct213 24061490 PMC3806542

[B3] Arceo-Gómez G. Ashman T.-L. (2011). Heterospecific pollen deposition: does diversity alter the consequences? *New Phytol.* 192 738–746. 10.1111/j.1469-8137.2011.03831.x 21777248

[B4] Arceo-Gómez G. Kaczorowski R. L. Patel C. Ashman T.-L. (2019). Interactive effects between donor and recipient species mediate fitness costs of heterospecific pollen receipt in a co-flowering community. *Oecologia* 189 1041–1047. 10.1007/s00442-019-04379-z 30877578

[B5] Arista M. Talavera M. Berjano R. Ortiz P. L. (2013). Abiotic factors may explain the geographical distribution of flower colour morphs and the maintenance of colour polymorphism in the scarlet pimpernel. *J. Ecol.* 101 1613–1622. 10.1111/1365-2745.12151

[B6] Ashman T.-L. Arceo-Gómez G. (2013). Toward a predictive understanding of the fitness costs of heterospecific pollen receipt and its importance in co-flowering communities. *Am. J. Bot.* 100 1061–1070. 10.3732/ajb.1200496 23624924

[B7] Benadi G. Pauw A. (2018). Frequency dependence of pollinator visitation rates suggests that pollination niches can allow plant species coexistence. *J. Ecol.* 106 1892–1901. 10.1111/1365-2745.13025

[B8] Benitez-Vieyra S. de Ibarra N. H. Wertlen A. M. Cocucci A. A. (2007). How to look like a mallow: evidence of floral mimicry between Turneraceae and Malvaceae. *Proc. R. Soc. B.* 274, 2239–2248. 10.1098/rspb.2007.0588 17623635 PMC2287375

[B9] Bergamo P. J. Susin Streher N. Traveset A. Wolowski M. Sazima M. (2020). Pollination outcomes reveal negative density-dependence coupled with interspecific facilitation among plants. *Ecol. Let.* 23 129–139. 10.1111/ele.13415 31650660

[B10] Binkenstein J. Renoult J. P. Schaefer H. M. (2013). Increasing land-use intensity decreases floral colour diversity of plant communities in temperate grasslands. *Oecologia* 173 461–471. 10.1007/s00442-013-2627-6 23568710

[B11] Briscoe A. D. Chittka L. (2001). The evolution of color vision in insects. *Ann. Rev. Entomol.* 46 471–510. 11112177 10.1146/annurev.ento.46.1.471

[B12] Briscoe Runquist R. Grossenbacher D. Porter S. Kay K. Smith J. (2016). Pollinator-mediated assemblage processes in California wildflowers. *J. Evol. Biol.* 29 1045–1058. 10.1111/jeb.12845 26864797

[B13] Bruno J. F. Stachowicz J. J. Bertness M. D. (2003). Inclusion of facilitation into ecological theory. *Trends Ecol. Evol.* 18 119–125. 10.1016/S0169-5347(02)00045-9

[B14] Burd M. Stayton C. T. Shrestha M. Dyer A. G. (2014). Distinctive convergence in Australian floral colours seen through the eyes of Australian birds. *Proc. R. Soc. B* 281:20132862. 10.1098/rspb.2013.2862 24573847 PMC3953836

[B15] Cavender-Bares J. Keen A. Miles B. (2006). Phylogenetic structure of Floridian plant communities depends on taxonomic and spatial scale. *Ecology* 87 S109–S122. 16922307 10.1890/0012-9658(2006)87[109:psofpc]2.0.co;2

[B16] Chittka L. (1997). Bee color vision is optimal for coding flower color, but flower colors are not optimal for being coded—Why? *Isr. J. Plant Sci.* 45 115–127. 10.1080/07929978.1997.10676678

[B17] de Jager M. L. Dreyer L. L. Ellis A. G. (2011). Do pollinators influence the assembly of flower colours within plant communities? *Oecologia* 166 543–553. 10.1007/s00442-010-1879-7 21170748

[B18] Diamond J. M. (1975). “Assembly of species communities,” in *Ecology and Evolution of Communities*, eds Cody M. L. Diamond J. M. (Cambridge, MA: Harvard University Press), 342–444.

[B19] Dyer A. G. Boyd-Gerny S. McLoughlin S. Rosa M. G. P. Simonov V. Wong B. B. M. (2012). Parallel evolution of angiosperm colour signals: common evolutionary pressures linked to hymenopteran vision. *Proc. R. Soc. B.* 279 3606–3615. 10.1098/rspb.2012.0827 22673351 PMC3396912

[B20] Earl J. B. Britt S. G. (2006). Expression of *Drosophila* rhodopsins during photoreceptor cell differentiation: insights into R7 and R8 cell subtype commitment. *Gene Expr. Patterns* 6, 687–694. 10.1016/j.modgep.2006.01.003 16495161

[B21] E-Vojtkó A. Bello F. Durka W. Kühn I. Götzenberger L. (2020). The neglected importance of floral traits in trait-based plant community assembly. *J. Veg. Sci.* 31 529–539. 10.1111/jvs.12877

[B22] Fargione J. Brown C. S. Tilman D. (2003). Community assembly and invasion: an experimental test of neutral versus niche processes. *Proc. Natl. Acad. Sci.* 100 8916–8920. 10.1073/pnas.1033107100 12843401 PMC166413

[B23] Feinsinger P. Busby W. H. Tiebout H. M. (1988). Effects of indiscriminate foraging by tropical hummingbirds on pollination and plant reproductive success: experiments with two tropical treelets (Rubiaceae). *Oecologia* 76 471–474. 10.1007/bf00377045 28312030

[B24] Fleishman L. J. Perez C. W. Yeo A. I. Cummings K. J. Dick S. Almonte E. (2016). Perceptual distance between colored stimuli in the lizard *Anolis sagrei*: comparing visual system models to empirical results. *Behav. Ecol. Sociobiol.* 70 541–555. 10.1007/s00265-016-2072-8

[B25] Freestone A. L. Harrison S. (2006). Regional enrichment of local assemblages is robust to variation in local productivity, abiotic gradients, and heterogeneity. *Ecol. Lett.* 9 95–102. 10.1111/j.1461-0248.2005.00849.x 16958873

[B26] Garcia J. E. Spaethe J. Dyer A. G. (2018). The path to colour discrimination is S-shaped: behaviour determines the interpretation of colour models. *J. Compar. Physiol. A* 203 983–997. 10.1007/s00359-017-1208-2 28866838

[B27] Ghazoul J. (2006). Floral diversity and the facilitation of pollination. *J. Ecol.* 94 295–304. 10.1111/j.1365-2745.2006.01098.x

[B28] Gumbert A. Kunze J. Chittka L. (1999). Floral colour diversity in plant communities, bee colour space and a null model. *Proc. R. Soc. Lond. Ser. B.* 266 1711–1716. 10.1098/rspb.1999.0836

[B29] Harris C. R. Millman K. J. van der Walt S. J. Gommers R. Virtanen P. Cournapeau D. (2020). Array programming with NumPy. *Nature* 585 357–362. 10.1038/s41586-020-2649-2 32939066 PMC7759461

[B30] Harrison S. Viers J. H. Quinn J. F. (2000). Climatic and spatial patterns of diversity in the serpentine plants of California. *Div. Distrib* 6 153–162. 10.1046/j.1472-4642.2000.00082.x

[B31] Horridge G. A. Mimura K. Tsukahara Y. (1975). Fly photoreceptors-II. Spectral and polarized light sensitivity in the drone fly Eristalis. *Proc. R. Soc. Londo. Ser. B. Biol. Sci.* 190 225–237. 10.1098/rspb.1975.0089 238210

[B32] Hubbell S. P. (2001). *The Unified Neutral Theory of Biodiversity and Biogeography.* Princeton, NJ: Princeton University Press.

[B33] Junker R. R. Blüthgen N. Brehm T. Binkenstein J. Paulus J. Martin Schaefer H. (2013). Specialization on traits as basis for the niche−breadth of flower visitors and as structuring mechanism of ecological networks. *Funct. Ecol.* 27 329–341. 10.1111/1365-2435.12005

[B34] Kemp J. E. Bergh N. G. Soares M. Ellis A. G. (2019). Dominant pollinators drive non-random community assembly and shared flower colour patterns in daisy communities. *Ann. Bot.* 123 277–288. 10.1093/aob/mcy126 29992277 PMC6344215

[B35] Koski M. H. Meindl G. A. Arceo-Gómez G. Wolowski M. LeCroy K. A. Ashman T.-L. (2015). Plant–flower visitor networks in a serpentine metacommunity: assessing traits associated with keystone plant species. *Arthr. Plant Interact.* 9 9–21. 10.1007/s11829-014-9353-9

[B36] Kraft N. J. B. Cornwell W. K. Webb C. O. Ackerly D. D. (2007). Trait evolution, community assembly, and the phylogenetic structure of ecological communities. *Am. Natur.* 170 271–283. 10.1086/519400 17874377

[B37] Kruckeberg A. (1984). The flora on California’s serpentine. *Fremontia (U.S.A.)* 11 3–10.

[B38] Leonard A. S. Dornhaus A. Papaj D. R. (2011). Flowers help bees cope with uncertainty: signal detection and the function of floral complexity. *J. Exp. Biol.* 214 113–121. 10.1242/jeb.047407 21147975 PMC2999516

[B39] Leonard A. S. Masek P. (2014). Multisensory integration of colors and scents: insights from bees and flowers. *J. Compar. Physiol. A* 200 463–474. 10.1007/s00359-014-0904-4 24710696

[B40] Lunau K. Fieselmann G. Heuschen B. van de Loo A. (2006). Visual targeting of components of floral colour patterns in flower-naïve bumblebees (*Bombus terrestris*; Apidae). *Naturwissenschaften* 93 325–328. 10.1007/s00114-006-0105-2 16568268

[B41] Maia R. Gruson H. Endler J. A. White T. E. (2019). pavo 2: new tools for the spectral and spatial analysis of colour in R. *Methods Ecol. Evol.* 10 1097–1107. 10.1111/2041-210X.13174

[B42] Makino T. T. Yokoyama J. (2015). Nonrandom composition of flower colors in a plant community: mutually different co-flowering natives and disturbance by aliens. *PLoS One* 10:e0143443. 10.1371/journal.pone.0143443 26650121 PMC4674055

[B43] McEwen J. R. Vamosi J. C. (2010). Floral colour versus phylogeny in structuring subalpine flowering communities. *Proc. R. Soc. B* 277 2957–2965. 10.1098/rspb.2010.0501 20484236 PMC2982023

[B44] Mesgaran M. B. Bouhours J. Lewis M. A. Cousens R. D. (2017). How to be a good neighbour: facilitation and competition between two co-flowering species. *J. Theoret. Biol.* 422 72–83. 10.1016/j.jtbi.2017.04.011 28419864

[B45] Meyer D. Zeileis A. Hornik K. (2020). *vcd: Visualizing Categorical Data*. R package version 1.4-8.

[B46] Mitchell R. J. Flanagan R. J. Brown B. J. Waser N. M. Karron J. D. (2009). New frontiers in competition for pollination. *Ann. Bot.* 103 1403–1413. 10.1093/aob/mcp062 19304814 PMC2701753

[B47] Moeller D. A. (2004). Facilitative interactions among plants via shared pollinators. *Ecology* 85 3289–3301. 10.1890/03-0810

[B48] Morales C. L. Traveset A. (2008). Interspecific pollen transfer: magnitude, prevalence and consequences for plant fitness. *Crit. Rev. Plant Sci.* 27 221–238. 10.1080/07352680802205631

[B49] Morehouse N. I. Rutowski R. L. (2010). In the eyes of the beholders: female choice and avian predation risk associated with an exaggerated male butterfly color. *Am. Natur.* 176 768–784. 10.1086/657043 20942644

[B50] Mori A. S. Furukawa T. Sasaki T. (2013). Response diversity determines the resilience of ecosystems to environmental change: response diversity and ecosystem resilience. *Biol. Rev.* 88 349–364. 10.1111/brv.12004 23217173

[B51] Muchhala N. Johnsen S. Smith S. D. (2014). Competition for hummingbird pollination shapes flower color variation in andean solanaceae: competition for pollination shapes flower color variation. *Evolution* 68 2275–2286. 10.1111/evo.12441 24766107

[B52] Ohashi K. Makino T. T. Arikawa K. (2015). Floral colour change in the eyes of pollinators: testing possible constraints and correlated evolution. *Funct. Ecol.* 29 1144–1155. 10.1111/1365-2435.12420

[B53] Ollerton J. Winfree R. Tarrant S. (2011). How many flowering plants are pollinated by animals? *Oikos* 120 321–326. 10.1111/j.1600-0706.2010.18644.x

[B54] Peitsch D. Fietz A. Hertel H. de Souza J. Ventura D. F. Menzel R. (1992). The spectral input systems of hymenopteran insects and their receptor-based colour vision. *J. Compar. Physiol. A* 170 23–40. 10.1007/bf00190398 1573568

[B55] Primante C. Dötterl S. (2010). A syrphid fly uses olfactory cues to find a non-yellow flower. *J. Chem. Ecol.* 36 1207–1210. 10.1007/s10886-010-9871-6 20924654

[B56] R Core Team (2019). *R: A Language and Environment for Statistical Computing.* Vienna: R Foundation for Statistical Computing.

[B57] Rathcke B. (1983). “Competition and facilitation among plants for pollination,” in *Pollination Biology*, ed. Real L. (New York, NY: Academic Press), 305–329. 10.1016/b978-0-12-583980-8.50019-3

[B58] Runquist R. B. Stanton M. L. (2013). Asymmetric and frequency-dependent pollinator-mediated interactions may influence competitive displacement in two vernal pool plants. *Ecol. Lett.* 16 183–190. 10.1111/ele.12026 23134452

[B59] Sargent R. D. Ackerly D. D. (2008). Plant–pollinator interactions and the assembly of plant communities. *Trends Ecol. Evol.* 23 123–130. 10.1016/j.tree.2007.11.003 18262307

[B60] SAS (2014). *Cary*. North Carolina: SAS Institute, Inc.

[B61] Schiestl F. P. Johnson S. D. (2013). Pollinator-mediated evolution of floral signals. *Trends Ecol. Evol.* 28 307–315. 10.1016/j.tree.2013.01.019 23480953

[B62] Shrestha M. Dyer A. G. Boyd-Gerny S. Wong B. B. M. Burd M. (2013). Shades of red: bird-pollinated flowers target the specific colour discrimination abilities of avian vision. *New Phytol.* 198 301–310. 10.1111/nph.12135 23368754

[B63] Shrestha M. Dyer A. G. Garcia J. E. Burd M. (2019). Floral colour structure in two Australian herbaceous communities: it depends on who is looking. *Ann. Bot.* 124 221–232. 10.1093/aob/mcz043 31008511 PMC6758583

[B64] Shrestha M. Lunau K. Dorin A. Schulze B. Bischoff M. Burd M. (2016). Floral colours in a world without birds and bees: the plants of Macquarie Island. *Plant Biol. J.* 18 842–850. 10.1111/plb.12456 27016399

[B65] van der Niet T. Johnson S. D. (2012). Phylogenetic evidence for pollinator-driven diversification of angiosperms. *Trends Ecol. Evol.* 27 353–361. 10.1016/j.tree.2012.02.002 22445687

[B66] Van Rossum G. Drake F. L. (2009). *Python 3 Reference Manual.* Scotts Valley, CA: CreateSpace.

[B67] Vorobyev M. Osorio D. (1998). Receptor noise as a determinant of colour thresholds. *Proc. R. Soc. B* 265 351–358. 10.1098/rspb.1998.0302 9523436 PMC1688899

[B68] Wakakuwa M. Kurasawa M. Giurfa M. Arikawa K. (2005). Spectral heterogeneity of honeybee ommatidia. *Naturwissenschaften* 92 464–467. 10.1007/s00114-005-0018-5 16136295

[B69] Waser N. M. (1986). Flower constancy: definition, cause, and measurement. *Am. Natur.* 127 593–603. 10.1086/284507

[B70] Webb C. O. (2000). Exploring the phylogenetic structure of ecological communities: an example for rain forest trees. *Am. Natur.* 156 145–155. 10.2307/3079215 10856198

[B71] Webb C. O. Ackerly D. D. McPeek M. A. Donoghue M. J. (2002). Phylogenies and community ecology. *Ann. Rev. Ecol. Syst.* 33 475–505. 10.2307/3069271

[B72] Wei N. Kaczorowski R. L. Arceo-Gómez G. O’Neill E. M. Hayes R. A. Ashman T.-L. (2020). Pollinator niche partitioning and asymmetric facilitation contribute to the maintenance of diversity. *Ecology* 10.1101/2020.03.02.974022

[B73] Wikstrom N. Savolainen V. Chase M. W. (2001). Evolution of the angiosperms: calibrating the family tree. *Proc. R. Soc. B* 268 2211–2220. 10.1098/rspb.2001.1782 11674868 PMC1088868

[B74] Wilcock C. Neiland R. (2002). Pollination failure in plants: why it happens and when it matters. *Trends Plant Sci.* 7 270–277. 10.1016/s1360-1385(02)02258-6 12049924

[B75] Wolf A. Brodmann P. A. Harrison S. (1999). Distribution of the rare serpentine sunflower, Helianthus exilis (Asteraceae): the roles of habitat availability, dispersal limitation and species interactions. *Oikos* 84 69–76. 10.2307/3546867

[B76] Wolowski M. Carvalheiro L. G. Freitas L. (2017). Influence of plant–pollinator interactions on the assembly of plant and hummingbird communities. *J. Ecol.* 105 332–344. 10.1111/1365-2745.12684

