# Supplementary figures and images for: Floral Color Properties of Serpentine Seep Assemblages Depend on Community Size and Species Richness
Source: Front Plant Sci. 2021 Jan 8;11:602951. doi: 10.3389/fpls.2020.602951 (PMC7820368; doi:10.3389/fpls.2020.602951)

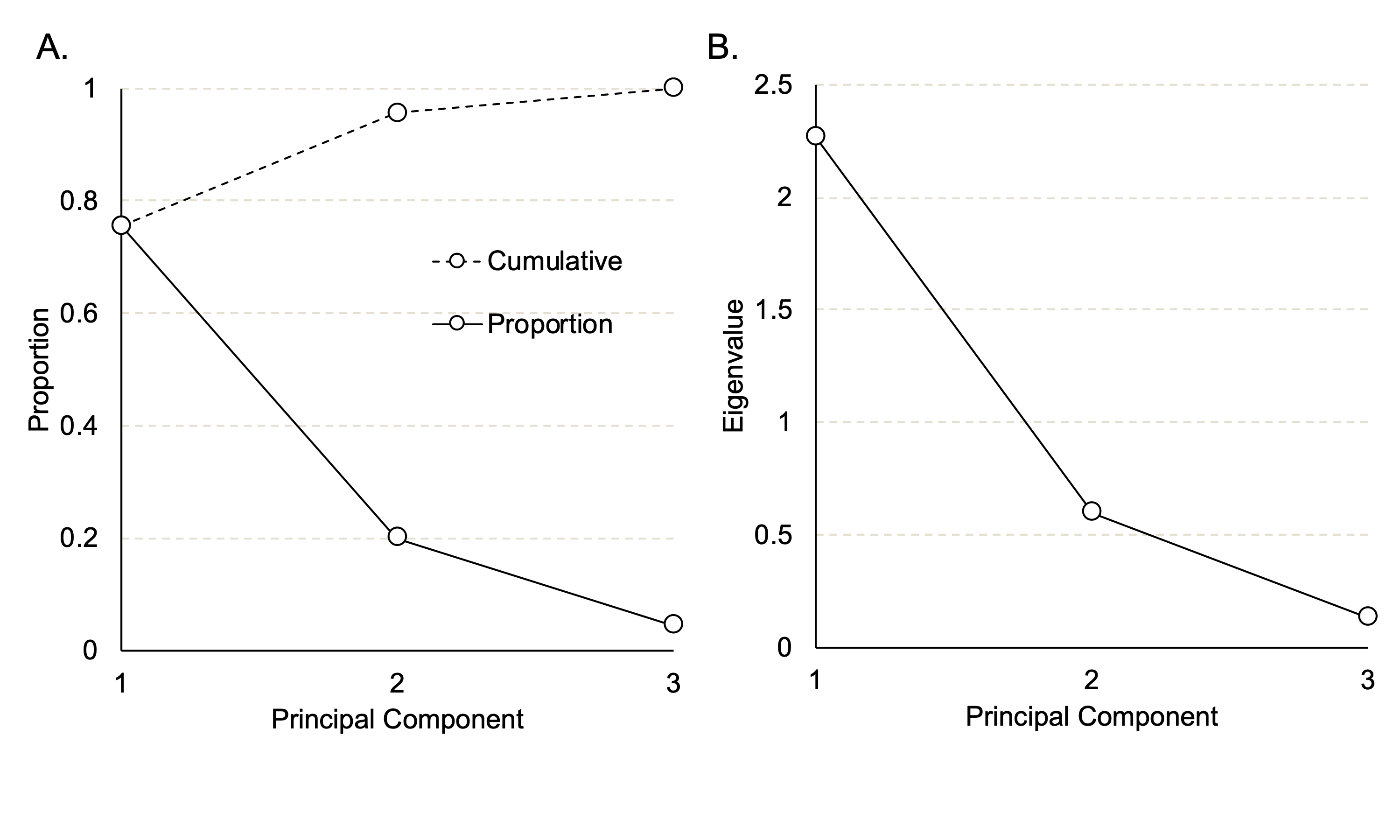

Supplement: Supplementary Figure 1 — Results of principal component analysis. (A) Proportion of variation explained by each Principal Component axis, with dashed line representing cumulative proportion, solid line representing proportion. (B) Eigenvalues of Principal Component Axes. [file Image_1.JPEG]

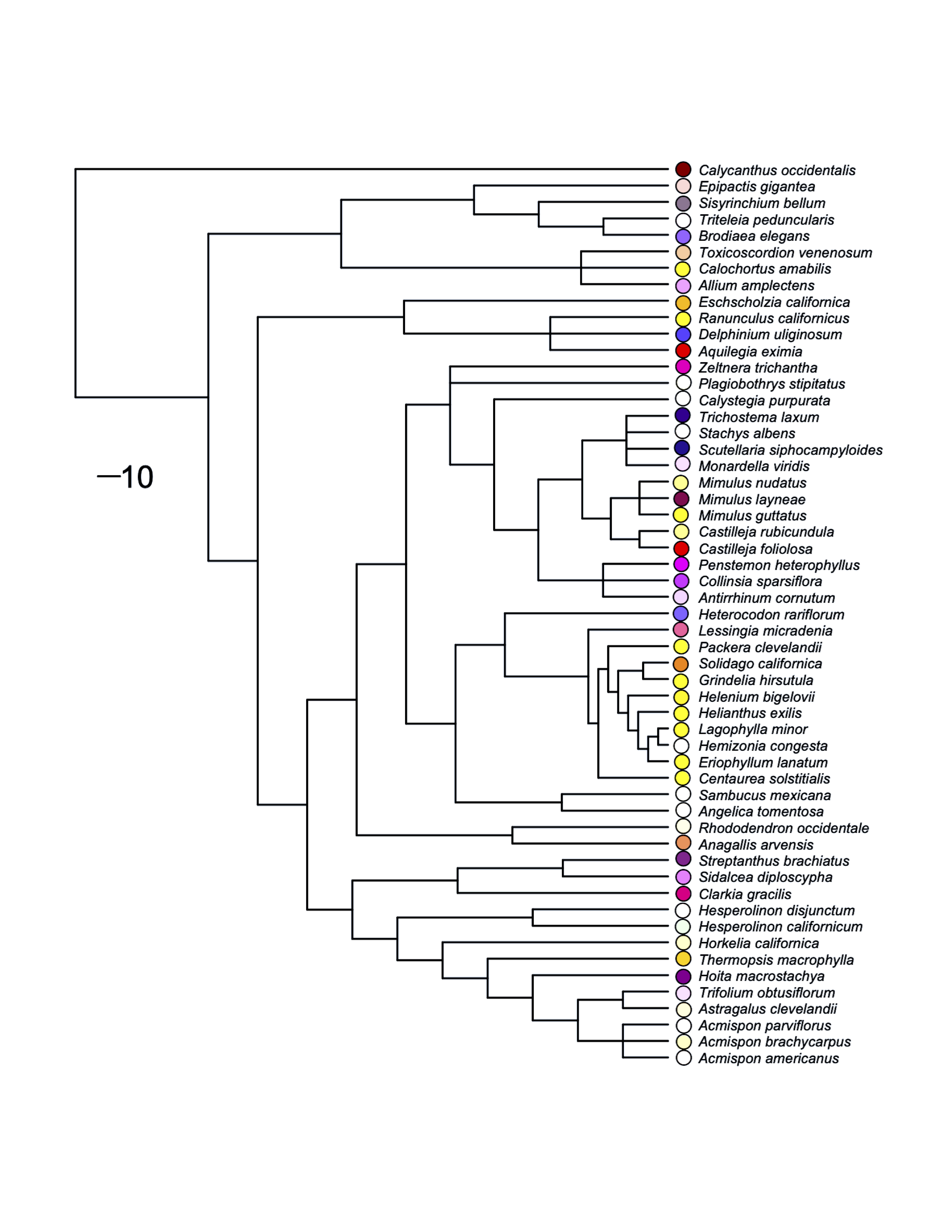

Supplement: Supplementary Figure 2 — Phylogenetic tree of 55 plant species used in study. Circles are colored to represent human vision interpretation of flower color. Branch length reference unit of 10 represents 10 Mya. [file Image_2.JPEG]
