# Supplementary material for: Floral Color Properties of Serpentine Seep Assemblages Depend on Community Size and Species Richness
Source: Front Plant Sci. 2021 Jan 8;11:602951. doi: 10.3389/fpls.2020.602951 (PMC7820368; doi:10.3389/fpls.2020.602951)
Supplement: Supplementary Methods 1 — Detail of color vision modeling equations used in methods. [file Presentation_1.PDF]

The difference in quantum catches q of photoreceptor i for two flower spectra (in our case, two co-flowering species) is:

$$\Delta q_i = q_i^1 - q_i^2$$

where

$$q_i^j = ln\left(\frac{Q_i^j}{Q_i^B}\right)$$

where photoreceptor output i for flower j is the natural log of its quantum catch divided by the quantum catch of the background (in our case, the foliage spectra). More specifically, the quantum catch for photoreceptor i of flower j between 300-700 nanometers (the typical range of visible spectra for insects) is:

$$Q_i^j = \int_{300}^{700} R(\lambda)\, I(\lambda) A_i(\lambda) d\lambda$$

where for a given wavelength λ, R is the reflectance spectrum, I is irradiance spectrum of illuminant, and A is the absorbance of photoreceptor i (Vorobyev and Osorio 1998).

The quantum catch of photoreceptor i for background B between 300-700 nanometers is:

$$Q_i^B = \int_{300}^{700} R^B(\lambda)\, I(\lambda) A_i(\lambda) d\lambda$$

where for a given wavelength λ, R^B is the reflectance spectrum of background, I is irradiance spectrum of illuminant, and A is the absorbance of photoreceptor i (Vorobyev and Osorio 1998).

As *A. mellifera* has three color photoreceptor types (Ultraviolet, Blue, and Green), we utilized a trichromatic color vision model. Using these differences in quantum catches of two flowers against background in a trichromatic color vision system, we estimated this color disparity (ΔS^t) as

$$\Delta S^t = \left[\frac{(e_1^2(\Delta q_3 - \Delta q_2)^2 + e_2^2(\Delta q_3 - \Delta q_1)^2 + e_3^2(\Delta q_1 - \Delta q_2)^2)}{((e_1 e_2)^2 + (e_1 e_3)^2 + (e_2 e_3)^2)}\right]^{1/2}$$

where e_i is the noise term for photoreceptor i (Vorobyev and Osorio 1998). This noise term is further defined as (Osorio 2004):

$$e_i = \left[\frac{\omega^2}{\eta_i} + \frac{2}{(Q_i^1 + Q_i^2)}\right]^{1/2}$$

where ω is the Weber fraction, η is the relative density of photoreceptor type i on the retina, and Q represents the quantum catch for photoreceptor type i for the floral spectra of a given species.

For *Eristalis tenax*, which has four color photoreceptor types (R7y, R8y, R7p, R8p), we used photoreceptor sensitivities provided by M. Shrestha and A.G. Dyer (pers. comm.). Using differences in quantum catches of two flowers against background in a tetrachromatic color vision system, we estimated color disparity as:

$$\Delta S^t = \left[\frac{\left((e_1 e_2)^2(\Delta q_4 - \Delta q_3)^2 + (e_1 e_3)^2(\Delta q_4 - \Delta q_2)^2 + (e_1 e_4)^2(\Delta q_2 - \Delta q_3)^2 + (e_2 e_3)^2(\Delta q_4 - \Delta q_1)^2 + (e_2 e_4)^2(\Delta q_3 - \Delta q_1)^2 + (e_3 e_4)^2(\Delta q_2 - \Delta q_1)^2\right)}{((e_1 e_2 e_3)^2 + (e_1 e_2 e_4)^2 + (e_1 e_3 e_4)^2 + (e_2 e_3 e_4)^2)}\right]^{1/2}$$

where e_i is the noise term for photoreceptor *i* (Vorobyev and Osorio 1998). This noise term is further defined as (Osorio 2004):

$$e_i = \left[\frac{\omega^2}{\eta_i} + \frac{2}{(Q_i^1 + Q_i^2)}\right]^{1/2}$$

where ω is the Weber fraction, η is the relative density of photoreceptor type *i* on the retina, and *Q* represents the quantum catch for photoreceptor type *i* for the floral spectra of a given species.
